# Investigation of *Echinococcus multilocularis* in Environmental Definitive Host Feces in the Asian and the European Parts of Turkey

**DOI:** 10.3389/fvets.2018.00048

**Published:** 2018-03-15

**Authors:** Ali Tümay Gürler, Francesca Gori, Cenk Soner Bölükbas¸, Şinasi Umur, Mustafa Açıcı, Peter Deplazes

**Affiliations:** ^1^Department of Parasitology, Faculty of Veterinary Medicine, Ondokuz Mayıs University, Samsun, Turkey; ^2^Department of Parasitology, Vetsuisse Faculty, University of Zurich, Zurich, Switzerland

**Keywords:** alveolar echinococcus, echinococcus multilocularis, fox, feces, Turkey

## Abstract

A study was carried out to investigate the presence of *Echinococcus multilocularis* in red foxes (*Vulpes vulpes*) in two regions of Turkey—central Anatolia (in Asia Minor) and Thrace (in the European part of Turkey). A total of 405 putative fox feces were collected from central Anatolia (186 specimens in 59 locations) and from Thrace (219 specimens in 114 locations). All samples were examined by the flotation and sieving method for taeniid eggs, and positive and putative samples were further analyzed by multiplex PCR. In seven samples from three locations in central Anatolia (5.1%) and in one (0.9%) from Thrace, *E*. *multilocularis* DNA was amplified, and this result was confirmed with another PCR specific for *E. multilocularis*. In addition, *Echinococcus granulosus* s.l. was found in two (0.5%) of the samples. Although alveolar echinococcosis (AE) is known as a serious zoonosis in Turkey, this is the first field study detecting *E. multilocularis* in collected fecal samples documenting the environmental contamination with eggs of this zoonotic parasite.

## Introduction

*Echinococcus multilocularis* is a taeniid parasite (Taeniidae, Cestoda) with a wild animal cycle that includes foxes and other canids as definitive hosts and rodents (particularly Arvicolidae) as intermediate hosts. The parasite is asymptomatic in definitive hosts but can cause a fatal disease, alveolar echinococcosis (AE), in intermediate and accidental hosts. *E. multilocularis* is a zoonotic cestode, and humans become infected by consumption of parasite eggs (1).

The geographic distribution of *E. multilocularis* depends on the presence of definitive hosts predating potential intermediate hosts. Central and eastern Europe, northern Asia, and North America are endemic for *E. multilocularis*, while some of these regions are regarded as highly endemic, namely, Central Europe, the Baltic countries, China, Japan, Mongolia, Kyrgyzstan, Russia, Turkey, and the western part of Alaska (2).

Although Turkey is considered a highly endemic region for human AE (2–4), there is little information available on the epidemiology of *E. multilocularis* in animal hosts. So far, two *E. multilocularis* infections in foxes, one from Trace (5) and the other from the eastern part of minor Asia (6), have been reported.

The aim of this study is to demonstrate the presence of *E. multilocularis* in field samples in two regions of Turkey: Central Anatolia where AE in humans is known to occur (7) and Thrace, where there is only historical evidence of possible endemicity.

## Materials and Methods

### Study Area

**Figure 1:**
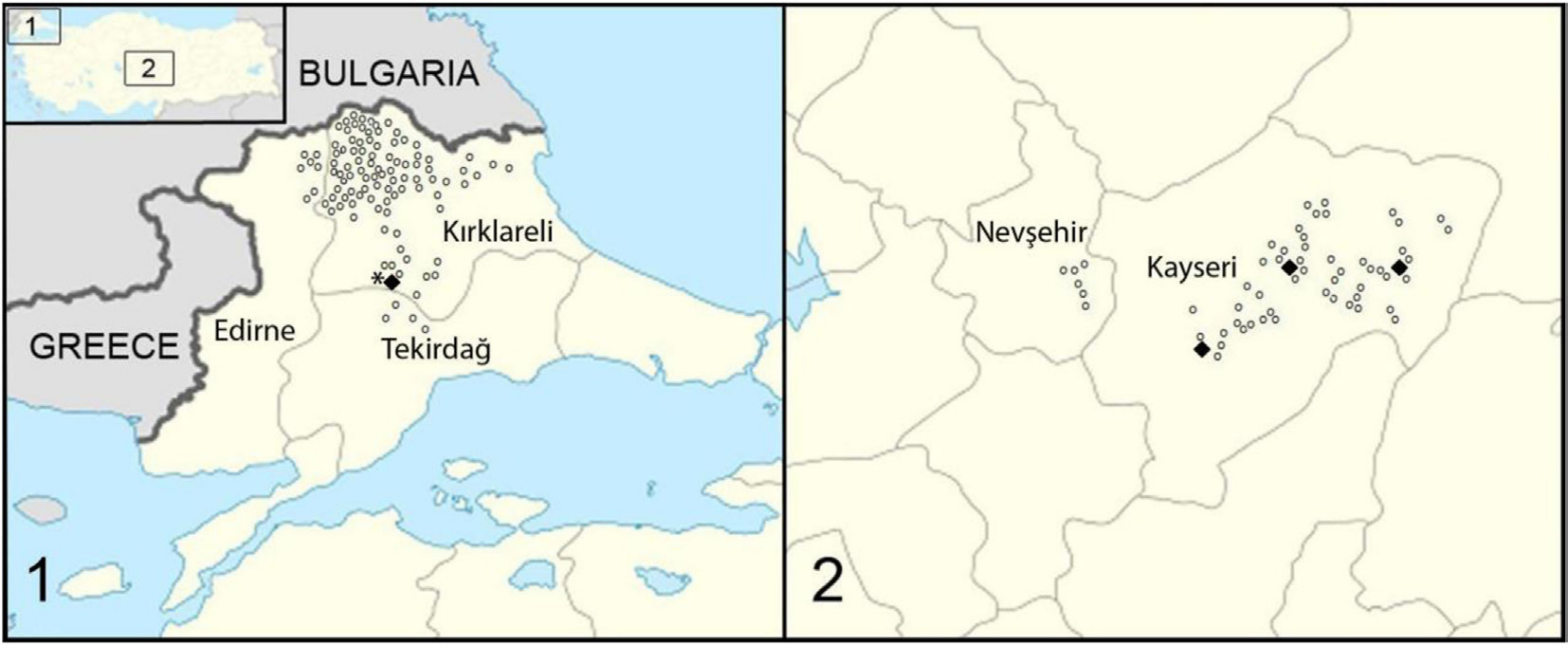
Study area and locations where fecal samples were collected in Turkey, (1) in Thrace and (2) in central Anatolia (Asia Minor). Open dots represent the location of samples negative for *Echinococcus multilocularis*; bold dots represent samples with positive *E*. *multilocularis* PCR result. The star localizes the origin of the fox infection with *E*. *multilocularis* found in the sixties (5).

Turkey is located on 36°–42° North and 26°–45° East longitude and consists of two parts—Anatolia (Asia Minor) in Asia and Thrace in Europe. The study was performed in five cities—two (Kayseri and Nevşehir) in central Anatolia and three (Kırklareli, Edirne, and Tekirdağ) in Thrace (Figure 1). There are no records on the fox population in Turkey. Consequently, fox feces were collected randomly from different locations, each at least 1 km distance from the other. All locations were situated in rural areas, including villages and fields with some locations around fox holes.

### Samples

Fox feces were collected in October 2014 in central Anatolia and in March 2015 in Thrace. They were identified based on their size, shape, and the presence of food residues such as hair, fruit, and feathers. The samples were picked up off the ground, put into sterile fecal containers and numbered, and the coordinates of each sample were recorded. In total, 405 samples were collected from 173 different locations; 186 specimens in 59 locations from central Anatolia and 219 specimens in 114 locations from Thrace.

### Diagnosis of *E. multilocularis*

All fecal samples were stored at −80°C for at least a week before analysis as safety precautions. Diagnosis of *E. multilocularis* consisted of three steps; determination of the presence of taeniid eggs in the samples; isolation of taeniid eggs; and molecular analysis of them.

All fecal samples were examined and taeniid eggs were concentrated by the flotation and sieving method, modified by Mathis et al. (8). Samples with identifiable taeniid eggs or with particles with similar size and shape as taeniid eggs and six negative samples collected in Thrace, where an *E. multilocularis* positive fox was identified in the 50 years previously (5), were further investigated by PCR. DNA was extracted by alkaline lysis (9) and amplified using the multiplex-PCR protocol according to Trachsel et al. (10). All positive samples for *E*. *multilocularis* were confirmed with the PCR protocol according to Stieger et al. (11).

In samples with *Echinococcus* spp. DNA amplification, the fox origin was confirmed by multiplex PCR as described by Nakao et al. (12). Positive control DNA extracted by tissue from the fox’s tongue and dog’s blood. Size of PCR products were 165 bp for fox and 355 bp for dog samples.

## Results

DNA was extracted from 60 of 405 putative fox fecal samples. *E. multilocularis* DNA was amplified in eight samples in both areas investigated (Table 1; Figure 1). From Anatolia, seven samples were found to be positive for *E. multilocularis*. Six of these samples contained other Cestoda spp. DNA. In one sample, *E. multilocularis, Echinococcus granulosus*, and non-*Echinococcus* spp. cestode DNA was amplified.

**Table 1:** Detection of *Echinococcus multilocularis* DNA in putative fox fecal samples collected in the environment in Turkey.

	Putative fox feces		Locations	
		
	Number of samples	Positive samples (%)	Number of locations	Positive locations (%)
Anatolia	186	7 (3.8)	59	3 (5.1)
Thrace	219	1 (0.5)	114	1 (0.9)
Total	405	8 (1.9)	173	4 (2.3)

In Thrace, *E. multilocularis* DNA was amplified in one of six egg negative samples. Furthermore, *E. granulosus* DNA was identified in one sample, and 25 samples contained DNA of non-*Echinococcus* cestodes.

All samples with *Echinococcus* spp. DNA amplification were identified by PCR as being of fox origin.

## Discussion

Turkey is a highly endemic region for *E. multilocularis*. Since 1939, when first reported, more than 750 human cases of AE have been recorded (3, 13–15), but the true incidence is estimated to be much higher, for example, Torgerson et al. proposed that there must be at least 100 cases of AE per year in Turkey (4). However, little information is available about the epidemiology of *E. multilocularis* in Turkey. There are no convincing reports on infections of wild rodents as intermediate hosts. In definitive hosts, only two cases have been published to date. One reports an *E. multilocularis* infection dating back to 1963 in which *E. multilocularis* was identified in a single necropsied red fox (*Vulpes vulpes*) from Thrace (5). Interestingly, further north, *E. multilocularis* has been described in rodents in Bulgaria and in foxes in Romania (16). In total, in this study, eight fox feces (1.9%) in four locations (2%) were positive for *E. multilocularis*. Three locations were from the central Anatolia and one from Thrace. Our finding of a positive fecal sample in Thrace, although negative for taeniid eggs, was confirmed by two PCRs targeting different mitochondrial genes. This therefore confirms that *E. multilocularis* is still endemic in Thrace in the same area where it was identified in 1960s. Interestingly, however, no cases of AE are known from this region, except possibly three suspect cases (17). By contrast, Anatolia (Asia Minor) is well known to be an endemic area for AE in humans, and recently the first infected fox has been documented (6). Between 1980 and 2010, 13 AE cases were reported from the city of Kayseri and one from the city of Nevşehir (7). The number of AE cases in humans increases gradually in an eastward direction in Turkey, with most cases recorded from East Turkey (17–19). In this study, we documented *E. multilocularis* in definitive hosts feces in central Anatolia for the first time. We amplified *E. multilocularis* DNA in 7 (4.4%) of 159 fox feces in Kayseri, but we found no evidence of infection in Nevşehir. This adds to information from neighboring countries. For example, in Iran, where only 40 AE cases have been reported in humans (20–22) even though *E. multilocularis* infection rates of 22.9% in foxes and of 20.9% in other carnivores (dog, jackal, hyena, and wolf) have been documented (23). In Iraq, by contrast, only two AE cases have been published, and no reports on definitive host infections with *E. multilocularis* are available (24).

In this preliminary study, we focused on the environmental contamination with *E*. *multilocularis* eggs based on fecal samples. However, we cannot exclude that the morphological identification of the samples was 100% specific for fox samples. Poulle et al. documented that the morphological assessment of fecal samples has a sensitivity of around 83% (95% confidence intervals, 61–95%) (25). Therefore, for all *Echinococcus* positive samples, the fox origin was confirmed by PCR even in the two samples with *E. granulosus*-specific amplification. Turkey is a well-known endemic area of *E. granulosus* (2), and *E. granulosus* prevalence in foxes ranged up to 50% in Australian foxes (26).

## Author Contributions

AG and PD developed the study protocol and oversaw all procedures. AG, FG, CB, MA, and ŞU organized to collection and investigation of the materials. AG and PD drafted the manuscript, and the final version was approved by all authors.

## Conflict of Interest Statement

The authors declare that the research was conducted in the absence of any commercial or financial relationships that could be construed as a potential conflict of interest.
